# Laboratory medicine in arterial hypertension

**DOI:** 10.11613/BM.2023.010501

**Published:** 2023-02-15

**Authors:** Merica Aralica, Vesna Šupak-Smolčić, Lorena Honović, Lucija Franin, Pavica Šonjić, Maja Šimac, Mihovil Horvat, Nina Poropat

**Affiliations:** 1 Clinical Department for Laboratory Diagnostics, Clinical Hospital Centre Rijeka, Rijeka, Croatia; 2 Department of Medical Informatics, Rijeka University School of Medicine, Rijeka, Croatia; 3 Department of Medical Biochemistry and Laboratory Medicine, General Hospital Pula, Pula, Croatia; 4 Department of Endocrinology, Diabetes and Metabolic Disorders, Clinical Hospital Centre, Rijeka, Croatia

**Keywords:** antihypertensive agent, clinical laboratory test, hypertension, medical laboratory science

## Abstract

In the initial diagnostics of arterial hypertension (AH) laboratory medicine is a cornerstone, along with a blood pressure (BP) measurement and an electrocardiogram. It mainly refers to routine blood and urine tests for diagnosis and monitoring primary hypertension and its associated conditions such as asymptomatic hypertension-mediated organ damage, chronic kidney disease and hypertensive disorders of pregnancy. In addition, long term non-fatal and fatal risks for cardiovascular (CV) events in hypertension are assessed based on clinical and laboratory data. Furthermore, laboratory medicine is involved in the management of hypertension, especially in monitoring the disease progression. However, antihypertensive drugs may interfere with laboratory test results. Diuretics, especially thiazides, can affect blood and urine sodium concentrations, or angiotensin-converting enzyme inhibitors and angiotensin receptor blockers can affect the blood biomarkers of the renin-angiotensin-aldosterone system (RAAS). It’s dysfunction plays a critical role in primary aldosteronism (PA), the most common endocrine disorder in secondary hypertension, which accounts for only small proportion of AH in relative terms but substantial proportion of hypertensives in absolute terms, affecting younger population and carrying a higher risk of CV mortality and morbidity. When screening for PA, aldosterone-to-renin ratio still contributes massively to the increased incidence of the disease, despite certain limits. In conclusion, laboratory medicine is involved in the screening, diagnosis, monitoring and prognosis of hypertension. It is of great importance to understand the preanalytical and analytical factors influencing final laboratory result.

## Introduction

In accordance with recent data published by the World Health Organization (WHO) about 41 million people around the world die from noncommunicable diseases (NCD) (*1*). On the list of the most prevalent NCD are cardiovascular diseases (CVD), respiratory diseases, cancer and diabetes along with arterial hypertension (AH) as a leading cause of premature death. According to WHO, about 1.28 billion people worldwide suffer from AH, including about 37% of Croatian adult population (*2*, *3*). With a prevalence of 30% to 45% among the adult population across the world, it represents the most common cause of doctor visits. It is well known that AH is a major independent risk factor for cardiovascular (CV) morbidity and mortality (*4*).

Considering the burden of AH on national and global healthcare and importance of the laboratory medicine in AH diagnosis and monitoring, the 33rd Annual Symposium „Laboratory medicine in arterial hypertension“ was organized under the auspices of the Croatian Society of Medical Biochemistry and Laboratory Medicine.

This descriptive review covers the presentations given at the symposium from clinical and laboratory approaches to AH, across the diseases and conditions associated with it and the influence of the antihypertensive drugs treatment on laboratory test results, to the screening for secondary hypertension (SH). In addition, an online survey was conducted with the aim to check symposium participants’ familiarity with the blood pressure (BP) measurement and methodology.

## Arterial hypertension – a practical approach

Hypertension is defined as a systolic pressure ≥ 140 mmHg and/or diastolic pressure ≥ 90 mmHg. The optimal arterial pressure value within the classification is defined as < 120/80 mmHg, a normal range is between 120 and 129 mmHg for systolic and/or 80 to 84 mmHg for diastolic pressure, whereas a high normal value is between 130 and 139 and/or 85 to 89 mmHg. Grade I of AH is a systolic pressure of 140-159 mmHg and/or a diastolic pressure of 90-99 mmHg; grade II includes a systolic pressure of 160-179 mmHg and/or diastolic pressure of 100-109 mmHg; grade III refers to a value > 180 mmHg for systolic and/or > 100 mmHg for diastolic pressure. Isolated systolic hypertension applies to people with systolic pressure > 140 mmHg and diastolic pressure < 90 mmHg (*4*).

The use of mercury-based blood pressure monitors (sphygmomanometers) is abandoned today within the European Union, and it is recommended that office arterial pressure measurement should be performed using automated oscillometric devices, which are validated and calibrated at least once a year. In addition to the BP measuring device, it is worth addressing the importance of the BP methodology. A patient’s body position and relaxation in accordance with environmental conditions can contribute to the accuracy of the BP measurement (*4*).

Continuous arterial pressure measurement has increasingly become the diagnostic standard, which is also indicated in the follow-up of patients with AH, re-evaluation of poorly controlled AH and assessment of treatment efficacy (*5*).

According to aetiology, hypertension can be primary (essential or idiopathic) (PH), which accounts for 85% of cases; and SH, which is present in up to 15% of patients. The most common causes of SH are chronic kidney disease (CKD) and endocrine disorders. Namely, AH can be a presentation of 15 different endocrine disorders, of which primary aldosteronism (PA) is the most common (*6*).

The diagnostic algorithm includes a thorough patient history and physical examination, focusing mainly on information about potential target organ damage and an increased CV and renal risk, and basic blood tests analysis with electrocardiogram (ECG) examination. It is recommended to assess the overall CV risk as well. Patients with hypertension and documented CVD, diabetes type 1 or 2, or CKD are considered to have a very high 10-year CV risk (> 10% mortality from CV causes) or high risk (5-10% mortality from CV causes) (*4*).

Treatment of AH includes non-pharmacological and pharmacological interventions. Non-pharmacological approaches refer to weight loss, reduction of salt, alcohol, and fat intake, and regular physical activity (*7*). Pharmacological therapy can be initiated using any available type of antihypertensive drugs like diuretics, angiotensin-converting enzyme (ACE) inhibitors, angiotensin receptor blockers (ARBs), beta-blockers and calcium channels blockers (CCBs). Among the drugs listed, ACE inhibitors and CCBs are the most commonly prescribed drugs in clinical practice.

## A survey on the blood pressure measurement and methodology

The BP measurement, like any other medical test, carries the risk of inaccuracy due to the use of an inadequate BP methodology or measurement devices. To overcome this risk, office BP measurement by a healthcare professional is still mandatory for the classification and management of AH (*4*).

Considering the widespread use of the BP measuring devices at home and in outpatient clinics and the many possible variants of BP methodology, we conducted an online survey of the knowledge among symposium participants (N = 103). The survey was based on the current guidelines for BP measurement in office (OBP) and out-of-office (*4*, *5*).

To the first question referring to the BP measuring devices, 69% (71/103) of the participants answered correctly that hybrid semi-automatic and automatic oscillometric devices are recommended for BP measurement. Apart from the measuring devices, the patient’s sitting position and body condition can also affect the BP values. Therefore, it is mandatory that the patient sits relaxed without talking for 3 to 5 minutes, before the test. According to the answers to the question about optimal body condition, the vast majority of participants (83%, 83/103) were familiar with the need to relax before BP measurement. In addition, a seated position with the back leaning against the chair’s back and with the legs uncrossed is preferred during the test. When asked about the position of the person’s legs, 94% (97/103) of participants responded correctly. In addition to rest, calm state and sitting position, there are other methodological factors that can influence BP values such as the position of the arm chosen for the test. The upper arm for BP measurement should be at heart level while the forearm is resting on table. If the upper arm is placed above the level of the right atrium, it may result in a false decrease in arterial pressure. Majority of the participants (72%, 74/103) were familiar with the influence of the upper arm position on the BP.

According to the guidelines, patients should avoid food consumption, exposure to cold, exercise, and abstain from cigarettes, caffeine and alcoholic beverages at least 30 minutes before the measurement. More than half of the participants (66%, 66/103) were aware of the need to abstain from coffee and cigarettes for the required period of time.

At home BP monitoring (HBPM) in the form of a diary is recommended as the best method for long-term monitoring of treated hypertension, and for detecting white-coat hypertension and masked hypertension. About 81% (83/103) of participants answered correctly that HBPM has the advantage over OBP measurement.

Blood pressure should be measured in the morning (before taking medication) and in the evening (before meal or taking medication if already prescribed) twice at 1-minute intervals over a 7 day period. When asked about measurement intervals, half of participants (52%, 54/103) knew about the HBPM protocol.

The device used for HBPM must be calibrated and the patient must be educated about self-measurement. When following these rules, there is no difference between the HBPM and OBP methods. Next question in the survey was whether there is a difference between the HBPM and OBM methods and 68% (70 /103) of participants chose the correct answer.

In general, values of BP measured by the patient at home are lower than those measured in the office. Therefore, the cut-off value indicating AH at HBPM is ≥ 135/85 mmHg, whereas in the office it is set at > 140/90 mmHg. According to the responses, only 18% (19/103) of the participants recognized differences in the cut-off values between the two types of BP measurements.

The survey results show an acceptable knowledge of the BP measurement among the participants, but there is also room for improvement.

## Laboratory diagnostics of arterial hypertension

In primary hypertension, the laboratory plays an important role in risk stratification for adverse CV and peripheral vascular events, in assessing asymptomatic hypertension-mediated organ damage (HMOD), and in determining the therapeutic approach for a patient (*4*).

According to the 2018 guidelines for the management of arterial hypertension issued by the European Society of Hypertension (ESH) and the European Society of Cardiology (ESC), the following data must be collected in every patient diagnosed at AH: detailed clinical history, physical examination, initial laboratory tests, and other diagnostic procedures (12-lead ECG) (*4*). The following routine laboratory tests are recommended at the initial evaluation: haemoglobin, haematocrit, glucose, haemoglobin A1c (HbA1c), creatinine, estimated glomerular filtration rate (eGFR) calculated according to the formula Chronic Kidney Disease-Epidemiology Collaboration (CKD-EPI), triglycerides, total cholesterol, low-density lipoprotein (LDL) cholesterol, high-density lipoprotein (HDL) cholesterol, potassium, sodium, uric acid, total protein, albumin, bilirubin, alkaline phosphatase, aspartate aminotransferase (AST), alanine aminotransferase (ALT) and gamma-glutamyltransferase (GGT). It is also recommended to perform a qualitative urine dip-stick test, microscopic examination, and albumin-creatinine ratio (ACR) in the spot urine sample (Table 1) (*4*).

**Table 1:** Routine laboratory tests for basic evaluation of arterial hypertension

**Sample type**	**Laboratory tests**
Blood	Complete blood count (haemoglobin, haematocrit) Lipid profile (triglycerides, total cholesterol, LDL cholesterol, HDL cholesterol) Electrolites (potassium, sodium) Liver function tests (total protein, albumin, bilirubin, alkaline phosphatase, AST, ALT, GGT) Metabolites (glucose, HbA1c, creatinine, uric acid) Equation (eGFR)
Urine	Qualitative urine dip-stick test and microscopic examination Albumin-creatinine ratio
LDL - low-density lipoprotein. HDL - high-density lipoprotein cholesterol. AST - aspartate aminotransferase. ALT - alanine aminotransferase. GGT - gamma-glutamyltransferase. HbA1c - haemoglobin A1c. eGFR - estimated glomerular filtration rate.	

Based on these laboratory test results, BP, other risk factors, asymptomatic HMOD, and comorbidities, patients should be evaluated for their 10-year risk of a fatal and nonfatal CV event. According to the 2021 ESC Guidelines on cardiovascular disease prevention in clinical practice, the 10-year risk is calculated using the Systematic Coronary Risk Estimation 2 (SCORE2) for patients aged 40-69 years or the Systematic Coronary Risk Estimation 2-Older Person (SCORE2-OP) for patients aged 70 years or older (*7*). Risk assessment is not required in patients already diagnosed with CVD, CKD (stage 3-5, eGFR < 59 mL/min/1.73m^2^), dyslipidemia (total cholesterol concentration > 8.0 mmol/L), and/or diabetes with or without complications. The latter two, together with hypertension, form a metabolic triad that multiplies the risk of a fatal CV event. Patients with these conditions are already in a very high or high risk category, so calculating the 10-year risk does not change their predicted risk (*7*). A 10-year risk assessment is particularly useful for patients without established comorbidities or other risk factors, as they may benefit from lifestyle intervention or therapy. Laboratory diagnostics play a direct role in the calculation of SCORE2 and SCORE2-OP because one of the variables used for risk calculation is non-HDL-cholesterol, which is calculated by subtracting HDL-cholesterol concentration from total cholesterol concentration (*7*). Unfortunately, SCORE2 and SCORE2-OP do not consider some additional risk factors that may alter the 10-year overall risk for CVD. One of the risk factors for CVD, independent of the presence of hypertension is uric acid. Its concentration above 360 µmol/L affects the classification of 10-year risk for CVD (*8*). Other laboratory tests that were considered significant have recently been shown to be less useful for patient risk stratification, such as high-sensitivity C-reactive protein (*9*). Further studies are needed to clarify the role of cardiac markers such as N-terminal pro-brain natriuretic peptide (NT-proBNP) and high-sensitivity troponin (hsTn) in reclassifying 10-year risk scores (*7*).

Asymptomatic HMOD has a significant impact on risk modification. Hypertension-mediated organ damage are structural or functional changes in target organs such as the heart, brain, retina, kidneys, and blood vessels. These changes are not included in the SCORE2 or SCORE2-OP mathematical model and are often not detected in time. In addition, there is a possibility of damage to multiple organs, which increases the risk. Detection of HMOD can change the 10-year risk from moderate to high or from low to moderate, which also changes the therapeutic approach. There are several diagnostic procedures to identify HMOD, and the laboratory is critical to determine the stage of CKD, as it is an independent risk factor for adverse CV events. eGFR values < 60 mL/min/1.73m^2^ or ACR > 3.4 mg/mmol indicate asymptomatic renal dysfunction (*10*).

In conclusion, AH is an independent risk factor for CVD. Laboratory diagnostics cannot reveal the cause of primary AH, but it can reveal other risk factors that, together with hypertension, have a direct influence on the therapeutic approach. The effect of therapy also becomes accessible via measurable laboratory parameters such as LDL-cholesterol, non-HDL-cholesterol or apolipoprotein B (*4*).

## Arterial hypertension and chronic renal disease

Chronic kidney disease affects nearly 11% of the adult population. It is a common clinical condition in developed countries with a significant public health burden, due to the numerous complications that occur during disease development (*11*).

After diabetes, AH or nephroangiosclerosis is the most common cause of CKD. However, if we take into account that one in two patients with diabetes has AH, it turns out that AH is the main cause of CKD. It is estimated that about 850 million people worldwide suffer from CKD, resulting in almost 2.4 million deaths *per* year. As reported by the Croatian registry, nearly 500 new patients in Croatia require dialysis or transplantation each year. According to the prevalence of hypertension (about 40%) and the number of adults (3,400,000), there are about 1,350,000 hypertensive patients in Croatia. In 2005, 102 patients in Croatia underwent dialysis because of hypertensive kidney disease which is one end-stage renal disease *per* 13,000 hypertensive patients (or 76 *per* 1,000,000). Despite the low incidence of end-stage renal disease in hypertensive patients, a significant number of renal failures occur because of the high prevalence of hypertension (*12*, *13*).

The definition of CKD includes decreased eGFR (< 60 mL/min/1.73m^2^) and/or renal impairment (histological abnormalities of the kidneys, structural abnormalities of the kidneys, history of kidney transplantation) accompanied by albuminuria (> 30 mg/day) for more than three months (*10*, *14*).

The grading of CKD is based almost exclusively on two laboratory parameters, eGFR (categories 1 to 5) and albuminuria (categories 1 to 3), and is used to predict the progression of the disease itself (*10*).

Chronic kidney disease is not an independent disease, but it has significant cause-and-effect relationships with a number of diseases, of which diabetes should be particularly emphasized (causing diabetic nephropathy), but also hypertension and atherosclerosis. The role and impact of AH should be reviewed from the perspective of traditional common risk factors for other cardiovascular events in patients with CKD. Arterial hypertension leads to changes in the endothelium of the renal blood vessels and increased permeability leading to the accumulation of macromolecules from the circulation that increases intraluminal pressure. To compensate for the increased intraluminal pressure, hypertrophy of the media occurs in a process called hypertensive nephrosclerosis (*15*).

There are two types of changes in the blood vessels of patients with CKD, namely atherosclerosis and arteriosclerosis. Atherosclerosis is characterized by atherosclerotic plaques on various medium-sized blood vessels, such as the femoral, coronary, and carotid arteries. Age and metabolic syndrome play an important role in the pathogenesis of atherosclerotic changes. Arteriosclerosis manifests as stiffening of the blood vessels that occurs with aging and occurs much earlier in patients with CKD. Thus, altered blood vessels lead to significant changes in haemodynamics. Calcifications in the blood vessels are more pronounced with the progression of chronic renal failure, especially media calcification. Disorders in mineral metabolism play a key role in the development of vascular calcification (*15*).

The development of CKD is favoured by general risk factors such as age, obesity, hyperuricaemia, dyslipidemia, tobacco use, family history and male gender, but also by risk factors for kidney damage such as albuminuria, anaemia, bone mineralization disorders, malnutrition, toxic metabolites, inflammation and oxidative stress, and AH. In patients with CKD, AH leads to more rapid progression of renal disease with CV complications such as cardiomyopathy, atherosclerosis, arterial stiffening, calcification and subsequent ischemic heart disease, heart failure, cerebrovascular and cardiovascular death (*15*, *16*).

In general, AH leads to myocardial and vascular remodelling and nephrosclerosis. In almost 90% of patients with eGFR < 30 mL/min/1.73m^2^, AH was confirmed. Even normal and high-normal BP (120-139/80-89 mmHg) increase the risk of developing CKD and cardiovascular changes, and the range of blood pressure values that can be considered ideal for patients with CKD is very narrow (*17*). The current 2021 Kidney Disease Improving Global Outcomes (KDIGO) guidelines recommend a target BP < 120 mmHg in patients with stable renal function, < 130/80 mmHg for renal transplant patients and a reduction of 24-hour mean arterial pressure in children according to the 50th percentile for the age, sex and height (*18*).

Laboratory diagnosis of hypertensive nephropathy is very similar or almost the same as for CVD. Therefore, renal failure and proteinuria are risk factors for diseases of the CV system. Proteinuria and decreased eGFR are only renal manifestations of generalized vascular disease (*5*).

Limiting salt intake (< 100 mmol/day or 5.8 g of table salt) is important in hypertensive patients and in patients with signs and/or symptoms of fluid overload. Smoking cessation is also of paramount importance and it is necessary to assist the patient in this process. These measures are immediately followed by tight control of BP. Poorly controlled BP progressively damages the kidneys and increases the risk of CV mortality. With this in mind, treatment should be intensive, with target values of < 125/75 mmHg for patients with diabetes and/or proteinuria and < 130/80 mmHg for patients without proteinuria. Achieving optimal BP in patients with CKD is often very challenging and involves non-pharmacological measures and pharmacological treatment but also on-going education and support from healthcare professionals (*5*, *11*, *19*).

## Diagnostics of hypertensive disorders in pregnancy

As described above, AH is involved in the progression of chronic diseases such as CVD and CKD but it can also occur in a physiological process such as pregnancy.

Hypertensive disorders in pregnancy are among the most common medical disorders in pregnancy with serious maternal and perinatal morbidity (*20*, *21*). There is some variation among current national and international clinical practice guidelines, but the most common classification includes four categories: gestational hypertension, chronic hypertension, chronic hypertension with superimposed preeclampsia and preeclampsia (*21*, *22*). Gestational hypertension is new-onset hypertension (≥ 140 / ≥ 90 mmHg) after 20 weeks of gestation without proteinuria or other signs of preeclampsia. It should be carefully monitored because it may develop into preeclampsia or future chronic hypertension (*20*, *23*). Chronic hypertension in pregnancy is AH which occurs before pregnancy or before 20 weeks of gestation and persists after delivery. It may develop into superimposed preeclampsia which is diagnosed on the basis of symptoms suggestive of preeclampsia. Preeclampsia should be suspected if BP increases and is resistant to treatment or if proteinuria increases (*23*).

Traditionally, preeclampsia has been defined as hypertension that develops after 20 weeks of gestation in previously normotensive women and is accompanied by proteinuria. Evidence of proteinuria was considered mandatory for the diagnosis, but recent guidelines have expanded the definition of preeclampsia, questioning the need for proteinuria for its diagnosis (*20*-*22*). In addition, several studies have reported that accepted standard cut-off values for proteinuria in pregnancy are based on limited data from small studies and not on evidence of the prognostic significance of maternal or perinatal complications (*24*).

Recently, preeclampsia has been redefined as a multisystem disorder with proteinuria or indicative kidney damage, impaired liver function, thrombocytopenia, pulmonary edema and neurologic complications (*20*). Evaluation of end-organ damage includes findings such as abnormally low platelet count (less than 100x10^9^/L), elevated serum creatinine and elevated liver transaminases (twice the upper reference limit). Pregnant women with preeclampsia may experience a new-onset generalized seizures, a condition known as eclampsia. A variant of preeclampsia with severe features is known as HELLP syndrome, an acronym for *in vivo* haemolysis, elevated liver enzymes and low platelet count. Typical symptoms include abdominal pain, nausea and vomiting, accompanied by headache and visual changes (*23*).

In clinical practice pregnant women are still tested for proteinuria to detect preeclampsia. Historically the golden standard for proteinuria has been measurement of protein excretion in 24-hour urine collection with a cut-off at ≥ 300 mg/24h. Most guidelines recommend urine dipstick testing for initial screening of proteinuria with 1+ as threshold (*21*). The dipstick test is inexpensive, easy to use, and provides rapid results, but with low sensitivity and specificity. Nevertheless, these qualitative results should be confirmed by quantitative testing. Collection of 24-hour urine is challenging, time consuming, inconvenient, and misleading if collected inaccurately (*25*-*27*). There are recommendations and practices for measuring the protein-to-creatinine ratio (PCR) in spot urine samples with cut-off values at ≥ 30 mg/mmol or ≥ 0.3 mg/mg (*21*).

The meta-analysis was performed using PCR results to detect significant proteinuria from 13 studies (*25*). These thresholds ranged from 0.13 to 0.5 mg/mg, with sensitivity ranging from 65% to 89% and specificity from 63% to 87%. On average, the optimal threshold ranged from 0.30 to 0.35 mg/mg with optimal sensitivity and specificity. The spot PCR is a convenient alternative and a reasonable rule-out test (*25*, *26*). There is insufficient evidence to support the use of ACR in this setting (*25*, *26*).

Finally, it is important to understand both the strengths and limitations of current diagnostics, to provide adequate diagnosis, monitoring and treatment and to establish future research goals.

## Hyponatremia in arterial hypertension

Sodium is the most abundant extracellular fluid cation in the human body and plays a central role in maintaining water distribution and osmotic pressure. Sodium concentration is regulated by the adrenal glands and kidneys where sodium is filtered and then reabsorbed throughout the nephrons (*28*). Despite the fine regulatory pathways of sodium concentration in blood and urine, there is a well-known association between high dietary sodium intake and hypertension (*2*). On the contrary, treatment with antihypertensive drugs may carry the risk of hyponatremia defined as a serum sodium concentration < 135 mmol/L. It is the most common disorder of water and electrolyte balance, occurring in about 30% of hospitalized patients. Depending on the time of onset, it can be acute or chronic and is characterized by weakness, nausea, confusion, and in severe cases, seizures, and coma. Depending on the plasma osmolality, hyponatremia can be hypoosmotic, hyperosmotic or isosmotic (*29*).

Hypoosmotic hyponatremia is the most common form. It is characterized by increased sodium loss relative to water loss with sodium lost through the skin, digestive tract, or kidneys, or by increased extracellular volume due to water retention, for example, in renal failure, heart failure, liver cirrhosis or nephrotic syndrome. Euvolemic hypoosmotic hyponatremia is characterized by an increase in total body water due to excessive water intake or impaired free water excretion. The most common cause is the syndrome of inappropriate secretion of the antidiuretic hormone (ADH) (*30*).

Hyperosmotic hyponatremia occurs when there is an increased amount of other dissolved osmotically active substances in the extracellular fluid. This causes sodium to shift into the cells or water to shift into the extracellular space to maintain osmotic balance, and occurs in severe hyperglycemia (*30*).

Isoosmotic hyponatremia or pseudohyponatremia refers to a falsely decreased sodium result in patients with hyperlipidemia or hyperproteinemia. In serum or plasma electrolytes are present only in the aqueous phase, but in hyperlipidemia or hyperproteinemia, the proportion of the aqueous phase in the sample is reduced. In the clinical laboratory, electrolytes are mostly analysed by the method of the indirect ion-selective electrode (ISE) that includes sample dilution. The aqueous portion of the sample is diluted to a greater ratio, so the result of sodium is falsely decreased. The solution to this problem is a direct ISE method because it does not dilute the samples and it is usually used on a blood gas analyser (*31*).

In patients with hyponatremia, the diagnostic protocol to determine the cause of hyponatremia begins with the measurement of glucose, total protein, and lipids. Values within the reference intervals exclude pseudohyponatremia and hyperosmotic hyponatremia. The osmolality of serum and urine is then measured. It is also necessary to measure urine sodium. It is recommended to take urine and serum sample at the same time. Serum osmolality < 275 mOsm/kg H_2_O indicates hypoosmotic hyponatremia. It is also necessary to assess the volume status of the patient. Finally, it is recommended to measure thyroid and adrenal hormones, whereas measurement of ADH is not recommended (*29*, *30*).

Among antihypertensive medication, thiazide diuretics, ACE inhibitors, ARBs and CCBs belong to the first line of therapy (*4*). Thiazide diuretics are often accompanied by hyponatremia as a side effect that occurs in the first weeks after initiation of therapy. However, cases of hyponatremia caused by other groups of antihypertensives have also been described in the literature (*32*-*35*).

Thiazide diuretics inhibit sodium-chloride cotransporter in the distal tubule, thus preventing the reabsorption of sodium and chloride from the tubule, and decreasing the reabsorption of water in the distal tubule. The reduced volume of extracellular fluid then leads to the secretion of ADH, so that water is reabsorbed in the collecting ducts (*36*, *37*).

Because thiazides do not affect the renal medullary concentration gradient, they cannot affect the reduction of water reabsorption, which further promotes hyponatremia (*38*). Hyponatremia caused by thiazide diuretics occurs in 14-30% of patients taking this type of medication. It occurs within a few days to two weeks after starting therapy, so it is important to monitor these patients regularly. Furthermore, thiazide-induced hyponatremia is more common in women, the elderly, people with low body weight, and people who already have impaired renal function (*38*, *39*). Typical laboratory findings include decreased plasma osmolality, increased urine osmolality and increased urine sodium in the presence of normal thyroid and adrenal function. In case of thiazide-induced hyponatremia, thiazide therapy should be discontinued (*37*).

In conclusion, hyponatremia is the most common electrolyte disorder and it is important to recognize and exclude pseudohyponatremia. A major cause of hyponatremia may be treatment with antihypertensive medication, particularly thiazide diuretics, which are prescribed to many patients with AH.

## Effect of antihypertensive therapy on laboratory results

Due to worldwide high prevalence of AH in general population, antihypertensive drugs are among most frequently prescribed medication around the world and also in Croatia (*40*).

There are several groups of antihypertensives available on the market which help regulate various organs and/or organ systems in the human body, including the renin-angiotensin-aldosterone system (RAAS) (ACE inhibitors, ARBs or aldosterone antagonists), the cardiovascular system (CCBs), the kidneys (diuretics), and the central nervous system (methyldopa, β-blockers) (*4*).

Most of these drugs are not selective and can cause adverse side effects and affect laboratory findings. For example, ACE inhibitors decrease serum ACE activity, a test used in sarcoidosis diagnosis. Diuretics can cause hyponatremia, hypokalemia, as well as hyperuricemia and could potentially lead to gout development (*41*). Aldosterone antagonists, in addition to hyperkalemia can affect steroid hormone levels and lead to fertility problems (*41*). Angiotensin receptor blockers interact with platelet thromboxane A2/prostaglandin H2 (TXA2/PGH2) receptors prolonging bleeding time and decreasing platelet activity (*42*). Calcium channels blockers cause serum hypocalcemia and hypoglycemia (*43*). Methyl-dopa, often recommended during pregnancy if needed, sometimes alters red blood cell membranes resulting in autoimmune haemolytic anaemia and a positive Coombs test *via* immune modulation, positive antinuclear antibodies and smooth muscle actin antibodies, which can remain positive for up to six months after the last dose administration (*43*, *44*).

Current ESC/ESH guidelines for the management of newly diagnosed arterial hypertension, recommend dual drug therapy with either an ACE inhibitor or ARB, and a thiazide diuretic or CCB (*4*). National guidelines from 2017 also recommend multi-drug therapy based on medical history and various states of organ dysfunction (*5*).

Because these medications are widely used and multi-drug therapy is common, it is necessary to be familiar with the pharmacokinetics, pharmacodynamics, and side effects of antihypertensive medications to correctly interpret laboratory results.

## Screening for secondary hypertension

As aforementioned earlier, SH is hypertension with known and potentially treatable causes affecting approximately 10-20% of patients (*45*).

Secondary hypertension should be screened in individuals with hypertension onset before puberty or before 30 years of age, in stable hypertensive patients with acute rise in blood pressure and in individuals with malignant hypertension. It is recommended that an accurate measurement of BP is performed along with a review of the patient’s diet and/or medication regimen before screening for SH (*46*). The most common causes of SH are CKD along with renovascular hypertension, obstructive sleep apnea (OSA) and primary aldosteronism (*47*).

Primary aldosteronism is a group of diseases that involve dysfunction of RAAS with an autonomous production of aldosterone, resulting in renin suppression, hypertension, cardiovascular damage and occasionally hypokalemia (*48*). Screening for PA should be performed in people with hypertension resistant to three or more antihypertensive drugs, with hypertension and hypokalemia or adrenal incidentaloma or OSA, with a family history of early onset of hypertension or CVD, and in hypertensive first-degree relatives of PA patients (*48*). In PA diagnostic workflow, the aldosterone-to-renin ratio (ARR) plays the role of a triage test followed by the diagnostic tests (*47*). The ARR preanalytical phase is complex due to several potentially confounding factors. These include patient’s status, medications in use and blood collection, transport and storage. It appears that the antihypertensive drugs play the most critical role in a suboptimal approach to screening (*49*). Furthermore, there are analytical issues that should be considered when measuring aldosterone and renin concentrations or plasma renin activity (PRA). Regarding aldosterone, analytical issues are relatively low concentration of aldosterone in blood compared to other steroids and the role of aldosterone conjugates in immunoassay performance (*50*). Latter commonly introduces certain bias at lower concentrations due to lack of selectivity and including aldosterone conjugates into analysis (*51*). The use of the liquid chromatography coupled to the tandem mass spectrometry (LC-MS/MS) for aldosterone measurement may overcome analytical bias and interference of other steroids or steroid based drugs. The PRA was a pioneer assay for renin measurement but has recently been replaced by immunoassays for renin concentration measurement (direct renin concentration, DRC). The latter has better stability and turnaround time, but PRA is not affected by estrogen like DRC (*49*). However, the poor correlation between PRA and DRC at low renin levels should not be neglected because it is due to different metrics used for assessment of renin nature (*50*).

In the postanalytical phase, an appropriate cut-off value of ARR is essential for accurate screening of PA. However, it should be emphasised that the ARR result of an individual may be strongly influenced by confounding preanalytical factors and the used methodology (*48*). However, the introduction of ARR into screening has contributed to an increase in the incidence of PA in recent decades.

## References

[1] World Health Organization. Noncommunicable diseases. Available from: https://www.who.int/news-room/fact-sheets/detail/noncommunicable-diseases#:~:text=Key%20facts,%2D%20and%20middle%2Dincome%20countries. Accessed August 24th 2022.

[2] World Health Organization. Hypertension. Available from: https://www.who.int/news-room/fact-sheets/detail/hypertension. Accessed November 16th 2022.

[3] Institute for public health of Osječko-baranjska county. Available from: https://www.zzjzosijek.hr/17-svibnja-svjetski-dan-hipertenzije. Accessed November 16th 2022.

[4] Williams B Mancia G Spiering W Agabiti Rosei E Azizi M Burnier M ESC Scientific Document Group. 2018 ESC/ESH Guidelines for the management of arterial hypertension. The Task Force for the management of arterial hypertension of the European Society of Cardiology (ESC) and the European Society of Hypertension (ESH). Eur Heart J. 2018;39:3021–104. 10.1093/eurheartj/ehy339 30165516

[5] Jelaković B Baretić M Čikeš M Dika Ž Fištrek Prlić M Jelaković A Practical guidelines for diagnosing arterial hypertension of the Croatian Society of Hypertension of Croatian Medical Association and the Working Group on Hypertension of the Croatian Cardiac Society. Cardiol Croat. 2017;12:413–51. 10.15836/ccar2017.413

[6] William FY Calhoun DA Lenders JWM Stowasser M Textor SC. Screening for Endocrine Hypertension: An Endocrine Society Scientific Statement. Endocr Rev. 2017;38:103–22. 10.1210/er.2017-00054

[7] Visseren FLJ Mach F Smulders YM Carballo D Koskinas KC Bäck M ESC Scientific Document Group. 2021 ESC Guidelines on cardiovascular disease prevention in clinical practice. Eur Heart J. 2021;42:3227–37. 10.1093/eurheartj/ehab484 34458905

[8] Borghi C Rosei EA Bardin T Dawson J Dominiczak A Kielstein J Serum uric acid and the risk of cardiovascular and renal disease. J Hypertens. 2015;33:1729–41. 10.1097/HJH.0000000000000701 26136207

[9] Lin JS Evans CV Johnson E Redmond N Coppola EL Smith N. Nontraditional Risk Factors in Cardiovascular Disease Risk Assessment: Updated Evidence Report and Systematic Review for the US Preventive Services Task Force. JAMA. 2018;320:281–97. 10.1001/jama.2018.4242 29998301 PMC13564044

[10] Kidney Disease: Improving Global Outcomes (KDIGO) Work Group. KDIGO clinical practice guideline for evaluation and management of chronic kidney disease. Kidney Int. 2013;3:S1–163.

[11] Čala S. Kronična bubrežna bolest i arterijska hipertenzija. Medicus. 2007;16:219–25.

[12] Brück K Jager KJ Dounousi E Kainz A Nitsch D Ärnlöv J Methodology used in studies reporting chronic kidney disease prevalence: a systematic review. Nephrol Dial Transplant. 2015;30:iv6–16. 10.1093/ndt/gfv131 26209739 PMC4514069

[13] Hrvatsko društvo za nefrologiju, dijalizu i transplantaciju Hrvatskog liječničkog zbora. Available from http://www.hdndt.org/index.html. Accessed August 24th 2022.

[14] Jones G. The New International Recommendations for Chronic Kidney Disease. Available from https://www.aacc.org/publications/cln/articles/2014/october/kidney-disease. Accessed August 24th 2022.

[15] Carracedo J Alique M Vida C Bodega G Ceprián N Morales E Mechanisms of Cardiovascular Disorders in Patients With Chronic Kidney Disease: A Process Related to Accelerated Senescence. Front Cell Dev Biol. 2020;8:185. 10.3389/fcell.2020.00185 32266265 PMC7099607

[16] Gansevoort RT Correa-Rotter R Hemmelgarn BR Jafar TH Heerspink HJ Mann JF Chronic kidney disease and cardiovascular risk: epidemiology, mechanisms, and prevention. Lancet. 2013;382:339–52. 10.1016/S0140-6736(13)60595-4 23727170

[17] Mancia G Fagard R Narkiewicz K Redon J Zanchetti A Böhm M 2013 ESH/ESC guidelines for the management of arterial hypertension: the Task Force for the Management of Arterial Hypertension of the European Society of Hypertension (ESH) and of the European Society of Cardiology (ESC). Eur Heart J. 2018;39:3021–104.30165516

[18] Kidney Disease: Improving Global Outcomes (KDIGO) Blood Pressure Work Group. KDIGO 2021 Clinical Practice Guideline for the Management of Blood Pressure in Chronic Kidney Disease. Kidney Int. 2021;99:S1–87. 10.1016/j.kint.2020.11.003 33637192

[19] Smyth A O’ Donnell MJ Yusuf S Clase CM Koon TK Canavan M Sodium intake and renal outcomes: a systematic review. Am J Hypertens. 2014;27:1277–84. 10.1093/ajh/hpt294 24510182

[20] Sutton ALM Harper LM Tita ATN. Hypertensive disorders in pregnancy. Obstet Gynecol Clin North Am. 2018;45:333–47. 10.1016/j.ogc.2018.01.012 29747734

[21] Scott G Gillon TE Pels A von Dadelszen P Magee LA. Guideline – similarities and dissimilarities: a systematic review of international clinical practice guidelines for pregnancy hypertension. Am J Obstet Gynecol. 2022;226:S1222–36. 10.1016/j.ajog.2020.08.018 32828743

[22] Sinkey RG Battarbee AN Bello NA Ives CW Oparil S Tita ATN. Prevention, diagnosis and management of hypertensive disorders of pregnancy: a comparison of international guidelines. Curr Hypertens Rep. 2020;22:66. 10.1007/s11906-020-01082-w 32852691 PMC7773049

[23] Wilkerson RG Ogunbodede AC. Hypertensive disorders of pregnancy. Emerg Med Clin North Am. 2019;37:301–16. 10.1016/j.emc.2019.01.008 30940374

[24] Fishel Bartal M Lindheimer MD Sibal BM. Proteinuria during pregnancy: definition, pathophysiology, methodology, and clinical significance. Am J Obstet Gynecol. 2022;226:S819–34. 10.1016/j.ajog.2020.08.108 32882208

[25] Morris RK Riley RD Doug M Deeks JJ Kilby MD. Diagnostic accuracy of spot urinary protein and albumin to creatinine ratios for detection of significant proteinuria or adverse pregnancy outcome in patients with suspected preeclampisa: systematic review and meta-analysis. BMJ. 2012;345:e4342. 10.1136/bmj.e4342 22777026 PMC3392077

[26] Côté AM Brown MA Lam E von Dadelszen P Firoz T Liston RM Diganostic accuracy of urinary spot protein:creatinine ratio for proteinuria in hypertensive pregnant women:systematic review. BMJ. 2008;336:1003–6. 10.1136/bmj.39532.543947.BE 18403498 PMC2364863

[27] Côté AM Firoz T Mattman A Lam EM von Dadelszen P Magee LA. The 24-hour urine collection: gold standard or historical practice? Am J Obstet Gynecol. 2008;199:e625. 10.1016/j.ajog.2008.06.009 18718568

[28] Guyton AC, Hall JE, editors. [Medicinska fiziologija]. 13th ed. Zagreb: Medicinska naklada; 2017. (in Croatian)

[29] Turk Wensveen T Krznarić Zrnić I Hauser G Crnčević Orlić Ž. Hiponatrijemija – dijagnostički i terapijski pristup. Medicina Flum. 2014;50:414–24.

[30] Spasovski G Vanholder R Allolio B Annane D Ball S Bichet D Clinical practice guideline on diagnosis and treatment of hyponatraemia. Eur J Endocrinol. 2014;170:G1–47. 10.1530/EJE-13-1020 24569125

[31] Zhang YV. Pseudohyponatremia. Available from: https://www.aacc.org/science-and-research/clinical-chemistry-trainee-council/trainee-council-in-english/pearls-of-laboratory-medicine/2014/pseudohyponatremia. Accessed August 24th 2022.

[32] Falhammar H Skov J Calissendorff J Nathanson D Lindh JD Mannheimer B. Associations Between Antihypertensive Medications and Severe Hyponatremia: A Swedish Population-Based Case-Control Study. J Clin Endocrinol Metab. 2020;105:e3696–705. 10.1210/clinem/dgaa194 32285124 PMC7451505

[33] Kim DR Cho JH Jang WS Kim JS Jeong KH Lee TW Severe Hyponatremia Associated with the Use of Angiotensin II Receptor Blocker/thiazide Combinations. Electrolyte Blood Press. 2013;11:56–9. 10.5049/EBP.2013.11.2.56 24627706 PMC3950227

[34] Das S Bandyopadhyay S Ramasamy A Prabhu VV Pachiappan S. A case of losartan-induced severe hyponatremia. J Pharmacol Pharmacother. 2015;6:219–21. 10.4103/0976-500X.171880 26816476 PMC4714391

[35] Jiang Y Cai W Masquelin ME Gordon K. An Unexpected Case of Lisinopril-Associated Severe Hyponatremia. Cureus. 2020;12:e9039. 10.7759/cureus.9039 32782860 PMC7410512

[36] Palmer BF Clegg DJ. Renal Considerations in the Treatment of Hypertension. Am J Hypertens. 2018;31:394–401. 10.1093/ajh/hpy013 29373638

[37] Nadal J Channavajjhala SK Jia W Clayton J Hall IP Glover M. Clinical and Molecular Features of Thiazide-Induced Hyponatrmeia. Curr Hypertens Rep. 2018;20:31. 10.1007/s11906-018-0826-6 29637415 PMC5893690

[38] Glover M Clayton J. Thiazide-Induced Hyponatraemia: Epidemiology and Clues to Pathogenesis. Cardiovasc Ther. 2012;30:e219–26. 10.1111/j.1755-5922.2011.00286.x 21884020

[39] Sardar GK Eilbert WP. Severe Hyponatremia Associated with Diuretic Use. J Emerg Med. 2015;48:305–9. 10.1016/j.jemermed.2014.09.054 25499401

[40] Medical Product Utilisation in Croatia in. 2020. Available from: https://halmed.hr/Novosti-i-edukacije/Publikacije-i-izvjesca/Izvjesca-o-potrosnji-lijekova/#Tablica_3. Accessed August 24th 2022. (In Croatian)

[41] Rang HP, Dale MM, Ritter JM, Moore PK, editors. [Farmakologija]. 1st ed. Zagreb: Golden Markering – Tehnička knjiga; 2005. (In Croatian)

[42] Suresh A Sanji N Kamath PM Devendrappa SL Hanumanthareddy SG Maniyar I A Pilot Study on the Effect of Angiotensin Receptor Blockers on Platelet Aggregation in Hypertensive Patients - A Prospective Observational Study. J Clin Diagn Res. 2016;10:FC14–6. 10.7860/JCDR/2016/21743.8881 28050394 PMC5198347

[43] Grigoriadis C Tympa A Liapis A Hassiakos D Bakas P. Alpha-methyldopa-induced autoimmune hemolytic anemia in the third trimester of pregnancy. Case Rep Obstet Gynecol. 2013;2013:150278. 10.1155/2013/150278 24175105 PMC3794618

[44] LiverTox: Clinical and Research Information on Drug-Induced Liver Injury. Bethesda (MD): National Institute of Diabetes and Digestive and Kidney Diseases; 2012-. Methyldopa. [Updated 2020 Jan 10]. Available from: https://www.ncbi.nlm.nih.gov/books/NBK548173/. Accessed August 24th 2022. 31643176

[45] Hirsch JS Hong S. The Demystification of Secondary Hypertension: Diagnostic Strategies and Treatment Algorithms. Curr Treat Options Cardiovasc Med. 2019;21:90. 10.1007/s11936-019-0790-8 31823067

[46] Charles L Triscott J Dobbs B. Secondary Hypertension: Discovering the Underlying Cause. Am Fam Physician. 2017;96:453–61. 29094913

[47] Williams TA Reincke M. Management of endocrine disease: Diagnosis and management of primary aldosteronism: the Endocrine Society guideline 2016 revisited. Eur J Endocrinol. 2018;179:R19–29. 10.1530/EJE-17-0990 29674485

[48] Funder JW Carey RM Mantero F Murad MH Reincke M Shibata H The Management of Primary Aldosteronism: Case Detection, Diagnosis, and Treatment: An Endocrine Society Clinical Practice Guideline. J Clin Endocrinol Metab. 2016;101:1889–916. 10.1210/jc.2015-4061 26934393

[49] Stowasser M Gordon RD. Primary Aldosteronism: Changing Definitions and New Concepts of Physiology and Pathophysiology Both Inside and Outside the Kidney. Physiol Rev. 2016;96:1327–84. 10.1152/physrev.00026.2015 27535640

[50] Rehan M Raizman JE Cavalier E Don-Wauchope AC Holmes DT. Laboratory challenges in primary aldosteronism screening and diagnosis. Clin Biochem. 2015;48:377–87. 10.1016/j.clinbiochem.2015.01.003 25619896

[51] Debeljak Ž Marković I Pavela J Lukić I Mandić D Mandić S Analytical bias of automated immunoassays for six serum steroid hormones assessed by LC-MS/MS. Biochem Med (Zagreb). 2020;30:030701. 10.11613/BM.2020.030701 32774123 PMC7394251

